# *PYGO2* is overexpressed in multiple myeloma patients with 1q21 amplification, driving plasma cell proliferation and cell cycle progression

**DOI:** 10.1038/s41417-026-01060-5

**Published:** 2026-07-11

**Authors:** Nicolas Thomas Iannozzi, Paola Storti, Rosanna Vescovini, Camilla Sitzia, Andrea Poletti, Valentina Franceschi, Giannalisa Todaro, Gabriella Sammarelli, Denise Toscani, Vincenzo Raimondi, Mattia Dessena, Oxana Lungu, Fazel Mohammadi, Matteo Scita, Matia Bernardi, Anna Benedetta Dalla Palma, Stefania Ricci, Bruno Lorusso, Costanza Anna Maria Lagrasta, Federico Quaini, Luca Agnelli, Gaetano Donofrio, Nicola Giuliani

**Affiliations:** 1 Department of Medicine and Surgery, University of Parma, Parma, Italy; 2 Department of Medical-Veterinary Science, University of Parma, Parma, Italy; 3 Hematology and BMT Unit, “Azienda Ospedaliero-Universitaria di Parma”, Parma, Italy; 4 “Istituto Nazionale dei Tumori” Foundation, Milan, Italy

**Keywords:** Gene expression analysis, Myeloma, Genetic vectors

## Abstract

Chromosome 1q21 copy number alterations (1q21+) are among the most frequent secondary events in multiple myeloma (MM), correlating with poor prognosis and therapy resistance. However, the molecular drivers of this lesion remain poorly defined. Here, we identify PYGO2, a Wnt/β-catenin co-activator located on 1q21, as a critical effector of MM progression. Analysis of primary CD138^+^ plasma cells and public datasets revealed that *PYGO2* expression is significantly increased in 1q21+ patients and correlates with chromosome 1q copy number. High *PYGO2* levels were associated with adverse cytogenetics and shorter progression-free and overall survival. Functional studies showed that *PYGO2* knockdown (KD) in 1q21+ cell lines impaired proliferation, induced cell cycle perturbation coupled with CCND1 downregulation and reduced H3K4me3 enrichment, while increasing Carfilzomib and Bortezomib sensitivity. Conversely, *PYGO2* overexpression enhanced cell growth and proteasome inhibitor resistance. RNA-seq profiling of KD cells revealed coordinated suppression of canonical Wnt/TCF target genes involved in proliferation and reshaped the replication stress and DNA damage response transcriptome, suggesting enhanced replication stress. RNA-seq profiling of KD cells also revealed suppression of oncogenic and immunomodulatory pathways, suggesting a broader role in tumor-microenvironment crosstalk. Overall, our data establish *PYGO2* as a previously unrecognized effector of 1q21-driven aggressiveness in MM and suggest that it represents a therapeutic vulnerability in high-risk 1q21+ MM patients.

## Introduction

Multiple myeloma (MM) is a hematologic malignancy characterized by the abnormal proliferation and accumulation of plasma cells (PCs) in the bone marrow (BM) [1]. MM cells present genetic and molecular aberrations, which are defined as primary and secondary genetic events [2]. Among the secondary cytogenetic alterations, copy number alteration (CNA) of the 1q21 region (1q21+) is one of the most frequent events [3, 4]. The incidence and copy number phenotype of the 1q21 region increase during the progression and relapse of the disease. In fact, additional copies of 1q21 can be detected in around 40% of smoldering (SMM) and newly diagnosed MM (NDMM) and around 70% of relapse-refractory MM (RRMM) [5, 6].

As a gold standard, 1q21+ can be detected by fluorescent in situ hybridization (FISH). Gain (three copies) or amplification [amp (four or more copies)] of the 1q21 region occurs as a progression event and provides a fitness advantage to a particular sub-clone over other populations, contributing to tumor progression and relapse [7]. Particularly, MM patients with four or more copies of 1q21 are associated with high-risk features and poor response to standard therapies [4, 8]. Hanamura et al. showed that patients with 1q21 amp at diagnosis tended to have a more aggressive clinical course than those with 1q21 gain [5]. Moreover, the presence of 1q21+ has also been correlated with a reduced response and/or resistance to proteasome inhibitors (PIs) treatment, including bortezomib (BTZ) and carfilzomib (CFZ) [8–10] in MM patients.

The 1q21 amplicon is known to contain several genes that have been suggested to drive disease aggressiveness in 1q21+ patients [11–15]. However, the exact genes driving the 1q21+ have not been fully characterized. Furthermore, the mechanisms underlying the high-risk disease progression associated with 1q21+ have not been identified. Nevertheless, the identification in the 1q21 region of druggable targets is an emerging unmet medical need in MM patients for a personalized approach.

For this reason, in our study, we initially analyzed the gene expression of CD138^+^ PCs obtained from MM BM aspirates. This analysis allowed us to identify a set of genes within the 1q21 region, with copy number-driven expression. Among the genes most expressed in patients with 1q21 CNA compared to controls, we identified and focused on *PYGOPUS2 (PYGO2)*, a transcriptional factor involved in the Wnt/β-catenin signaling pathway. Across multiple solid malignancies, including glioma, prostate, liver, lung and breast cancer, *PYGO2* is frequently overexpressed and correlates with enhanced proliferation, epithelial-mesenchymal transition and adverse clinical outcome [16–18]. Particularly, in prostate cancer, *PYGO2* has been found to drive disease progression and metastasis [19].

The PYGO2 protein, composed of 406 amino acids with a molecular mass of 41 kDa, has two conserved domains, an N-terminal homology domain (NHD) and a C-terminal plant homology domain (PHD) zinc finger motif. Studies carried out in central nervous system tumors showed that deletion of the NHD domain is associated with 50% reduction of transcriptional activity [20]. The PHD domain has an important role in transcriptional activation, since PYGO2 is implicated in chromatin remodeling and in binding to methylated residues on lysine 4 of histone H3 (H3K4me3) [21]. The plant PHD finger is responsible for the binding, through adaptor proteins, to the N-terminal domain of β-catenin [21].

Particularly, PYGO2 is a nuclear protein involved in the TCF/β-catenin transcriptional complex, and it was first identified as a significant component of the canonical Wnt/β-catenin pathway, forming a linear chain with β-catenin *via* BCL9. This chain anchors β-catenin in the nucleus and activates transcription of target genes in the Wnt/β-catenin pathway [22, 23]. The target genes regulated by *PYGO2* are Cyclin D1 (*CCND1*), C-myc, Cyclin A, CD44 and multidrug resistance 1 (*MDR1/ABCB1*), which all play important roles in tumorigenesis, tumor progression, and prognosis. This emphasizes the implication and relevance of the PYGO2 protein in cell growth and proliferation [18, 24–26].

Despite this, the role of *PYGO2* in MM remains unknown. Here, we evaluated the expression profile of *PYGO2* in primary PCs of 1q21+ MM patients, and we characterized its functional roles in MM in vitro.

## Methods

### Purification of primary CD138^+^ BM PCs

We analyzed a total of 72 purified BM CD138^+^ PCs from a cohort of MM patients. Of the 72 patients, 29 (11 SMM and 18 NDMM) are part of our previously published GSE227907 database [27]. The remaining patients, used as a validation set, are 24 NDMM and 19 RRMM, with a median age of 75 years and a range of 41–89 (Table S1). All participants in the study provided their written consent in accordance with the Declaration of Helsinki. The Ethics Committee of the Hospital of Parma approved all study protocols.

Mononuclear cells (MNCs) were obtained from BM aspirates using Ficoll-Hypaque density sedimentation (Bichrome AG, Berlin, Germany). CD138^+^ MM cells were isolated by the immunomagnetic method using anti-CD138 monoclonal antibody-coated microbeads (MACS, Miltenyi Biotech, Bergisch-Gladbach, Germany). Only samples with >90% purity (flow cytometry analysis) were used.

### CoMMpass database analyses

Publicly available data from the Multiple Myeloma Research Foundation CoMMpass Study (release IA22) were downloaded in March 2025 from the Research Gateway portal (https://research.themmrf.org/). Baseline BM aspirates with both whole-genome CNA segmentation files and RNA-seq gene counts files, as well as complete clinical annotations, were retained, yielding 847 unique patients. All genomic coordinates refer to GRCh38.

Raw segmented log2 copy number ratios were corrected for purity using ABSOLUTE v1.0.6 [28] and for baseline region bias using BOBAFIT v1.0 [29]. Gene coordinates of genes relevant for MM biology and prognosis located on chromosome 1q (*PYGO2*, *CKS1B*, *BCL9* and *MCL1*) were intersected with segments to derive the genes' CN deviations from baseline (CN_dev). Continuous CN_dev values were converted to discrete calls (deletion ≤ −0.2, neutral > −0.2 and < 0.2, gain ≥ 0.2, amp ≥ 1.2). Spearman correlations between CN_dev values of 1q genes and expression values were calculated.

Raw RNA-seq gene counts provided by the CoMMpass consortium were normalised with the variance stabilising transformation in DESeq2 v1.42 [30] using the vst function. For each gene, quartiles were computed; the top quartile (Q1) defined high expression, while Q2–Q4 were combined as the reference group.

Overall survival (OS) and progression-free survival (PFS) were measured from diagnosis to death or progression; patients without an event were censored at last follow-up. Kaplan–Meier curves were produced using survival v3.5.5 and survminer v0.4.9 R packages and compared with two-sided log-rank tests. Multivariable Cox proportional hazards models were used to compute Hazard Ratios (HR), reported with 95 percent confidence intervals.

Binary matrices denoting common cytogenetic and mutational events were compiled: gain 1q (*CKS1B*), amp 1q, del 17p (*TP53*), del 13q (*RB1*), del 1p (*CDKN2C*), gain 8q (*MYC*), hyperdiploidy, t(4;14), t(11;14) and pathogenic *TP53* single-nucleotide variants or indels. Frequencies in the *PYGO2* Q1 group were compared with those in Q2–Q4 using Fisher's exact tests. Resulting *p* values were adjusted by the Benjamini–Hochberg method; a false discovery rate below 0.05 was considered significant. All analyses were executed in R v 4.3.2.

### Cancer dependency analysis (DEPMAP portal)

Gene dependency of cell lines, gene expression, and copy number data for *PYGO2* were obtained from the DepMap Portal Public 24Q4 release, downloaded in May 2025. The dependency data set used was CRISPR knockout gene effect scores computed with the Chronos model.

MM cell lines were identified by using the DepMap cell line selector, by setting the Disease Subtype filter to “Plasma Cell Myeloma”. This selection yielded a total of 34 MM cell lines, while the remaining 1159 tumor cell lines served as the non-MM reference cohort.

Spearman's rank correlation coefficients were calculated between *PYGO2* Chronos scores, expression and copy number values in the MM cell lines. A one-tailed Wilcoxon rank-sum test was used to compare the *PYGO2* Chronos scores between MM cell lines and other cancer types, as we hypothesized that MM cell lines have lower dependency scores compared to other cancer types. A two-tailed Wilcoxon rank-sum test was used to assess whether there is a significant difference in expression levels between MM cell lines and other cancer types. All analyses were conducted in R v4.3.2.

### *PYGO2* Knock-down and *PYGO2* Overexpression

*PYGO2* knockdown in JJN3, OPM2 and U266 was obtained by infection with a pGFP-C-shLenti lentiviral vector anti-*PYGO2* scramble (Scrb) and anti-*PYGO2* (sh*PYGO2*) (TL317702B, Origene). The sequence used for sh*PYGO2* was: 5’ CCTGCATACTCACATCTGACGGAGTTTGC 3’. *PYGO2* overexpression (OE) in OCI-MY5 was obtained by infection with ORF lentiviral vector, LV[Exp]-CMV>h*PYGO2*[NM_138300.4]:IRES:mCherry:T2A:Puro (241205-1107qbzVectorBuilder). Recombinant lentivirus was produced by transient transfection of 293T cells following the standard protocol. Viral supernatant was collected after transfection, concentrated by centrifugation at 26,000 rpm, and resuspended in cell growth medium. JJN3, OPM2, U266 and OCI-MY5 cells were seeded at 1 × 10^6^ cells/1 mL. All cell lines underwent the same puromycin selection (2 μg/ml) to minimize potential selection-related effects. The efficiency of the infection was assessed by flow cytometry using the GFP percentage for JJN3, OPM2 and U266, whereas mCherry was used for OCI-MY5. Stably transfected HMCLs were maintained in RPMI medium containing 20% FBS with 2 μg/ml puromycin until use.

### RNA-sequencing and bioinformatics analysis

Universal Plus mRNA-Seq with NuQuant kit (Tecan Genomics, Redwood City, CA) has been used for library preparation following the manufacturer’s instructions (library type: fr-secondstrand). RNA samples were quantified and quality tested by Agilent 2100 Bioanalyzer RNA assay or TapeStation RNA assay (Agilent Technologies, Santa Clara, CA). Final libraries were checked with Qubit 3.0 Fluorometer (Invitrogen, Carlsbad, CA) and Agilent Bioanalyzer DNA assay.

Libraries were prepared for sequencing and sequenced on paired-end 150 bp mode on NovaSeq 6000 (Illumina, San Diego, CA).

The number of reads (in millions) produced for each sample is listed in the table below.

**Table:** 

Library_ID	Obt Reads
ID3347_1-Scrb1	244.06
ID3347_2-Scrb2	252.04
ID3347_3-Scrb3	221.36
ID3347_4-sh*PYGO1*	211.12
ID3347_5-sh*PYGO2*	175.42
ID3347_6-sh*PYGO3*	202.03

Raw sequencing data were processed following a standard bioinformatics pipeline. Base calling, demultiplexing, and adapter masking were performed using Illumina BCL Convert v3.9.31. Adapter sequences were masked during demultiplexing, with masked regions converted to “N” characters and the corresponding base quality scores set to 2 to facilitate subsequent trimming. Quality filtering and adapter trimming were carried out using ERNE. Reads were aligned to the human reference genome (hg38) using STAR3 (default parameters), a splice-aware aligner optimized for RNA-Seq data that identifies splice junctions and maps reads accordingly. Transcript quantification was performed with StringTie4 (default parameters), which reconstructs full-length transcripts, including multiple splicing isoforms, and provides gene- and transcript-level abundance estimates.

Differential expression analysis was conducted using DESeq2, which fits a Generalized Linear Model (GLM) for each gene to compare expression levels across conditions. DESeq2 employs shrinkage estimation for dispersions and fold changes, enhancing the stability and interpretability of the results. This approach allows for a more quantitative assessment of differential expression, focusing on effect size rather than mere presence. Normalization was performed using the median-of-ratios method, and statistical significance was assessed using the Wald test.

Regarding Gene Sets Enrichment Analysis (GSEA), we downloaded the Molecular Signatures Database (MsigDB). To ensure the credibility of the analysis results, we selected 10,000 permutations in the software. A significant Hallmark gene set was screened based on normalized enrichment score (NES), while screening was based on normalized *p*-value < 0.05 and false discovery rate (FDR) < 25%.

### Statistics

Data were expressed as means ± standard deviation (SD). A two-tailed paired or unpaired Student’s test was used to test significant differences. The patients’ mRNA expression levels were compared using the Mann-Whitney independent t-test. Correlation coefficients were calculated using the Spearman rank correlation method. The two-way ANOVA was used to analyze the cell cycle assay. In all cases, *p* < 0.05 was considered significant. GraphPad Prism 8TM (GraphPad Software Inc., La Jolla, CA, USA) was used for all the statistical analyses.

A detailed version of the entire Materials and Methods section is available in the Supplementary Material.

## Results

### *PYGO2* is overexpressed in 1q21 + MM patients and correlates with worse survival

**Fig. 1:**
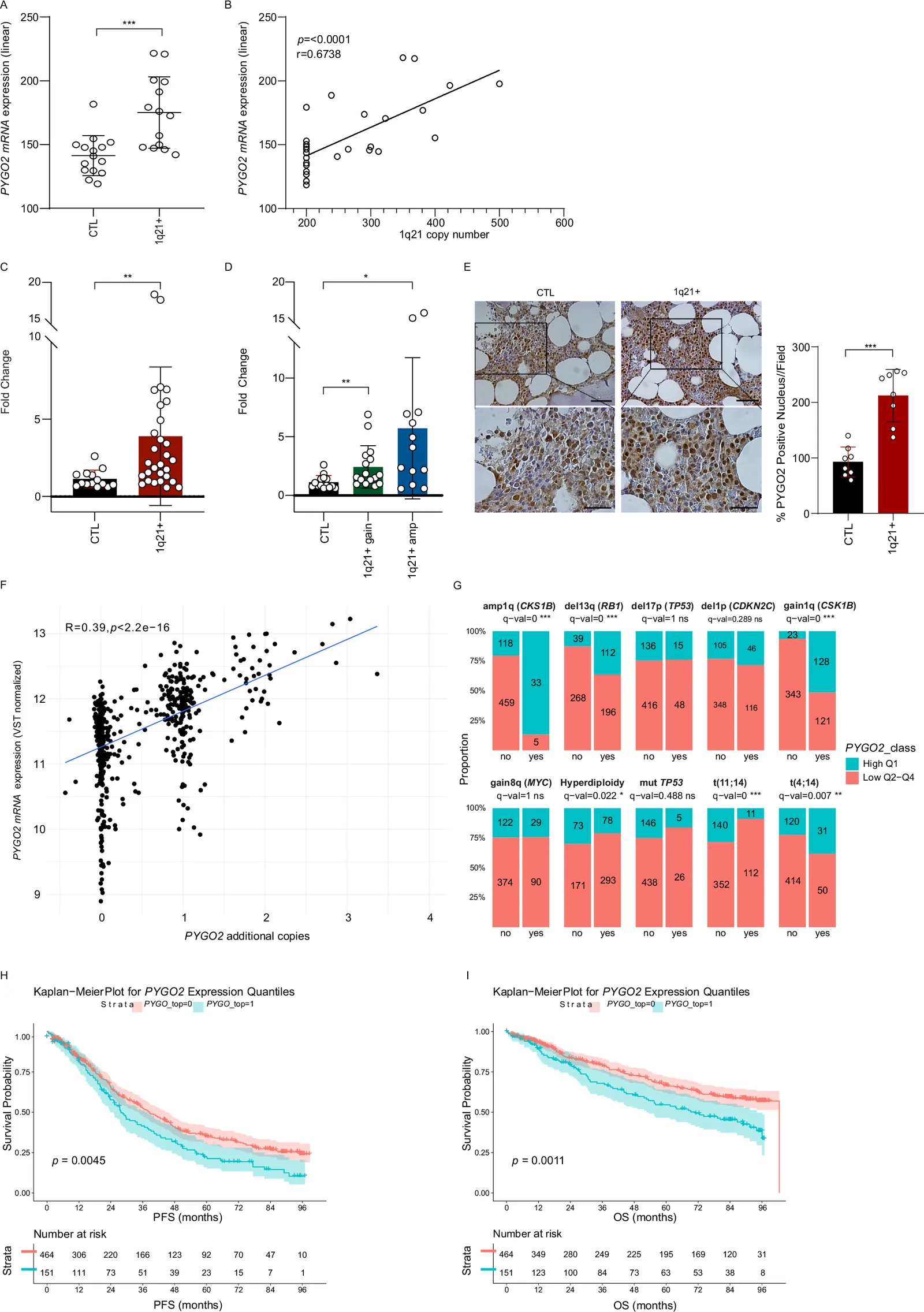
*PYGO2* is overexpressed in 1q21 + MM patients and correlates with worse survival. **A** *PYGO2* mRNA expression in primary PCs of 1q21+ MM patients (*n* = 14) compared to the control (CTL) (1q21 wild type) group (*n* = 15) on Gene CHIP arrays analysis. Statistical significance (***p < 0.001) was determined by a two-tailed non-parametric t-test (Mann-Whitney). **B** The scatter plot shows the relationship between *PYGO2* mRNA expression and 1q21 copy number in primary PCs of MM patients (*n* = 29), based on Gene CHIP array analysis. A Spearman correlation analysis revealed a significant positive monotonic association between *PYGO2* expression and 1q21 copy number (r = 0.6738, *p* < 0.0001). **C** Fold change of *PYGO2* mRNA normalized to the control gene *B2M* in primary PCs of 1q21+ MM patients (*n* = 29) compared to the CTL group (*n* = 14) by qRT-PCR. Statistical significance (**p < 0.01) was determined by a two-tailed non-parametric t-test (Mann-Whitney). **D** Fold change of *PYGO2* mRNA normalized to the control gene *B2M* in 1q21+ gain MM patients (*n* = 16) and 1q21+ amp MM patients (*n* = 13) compared to CTL group (*n* = 14) by qRT-PCR. Statistical significance (**p < 0.01) was determined by a two-tailed non-parametric t-test (Mann-Whitney). **E** PYGO2 protein stain (brownish) in BM PCs in 1q21+ and CTL patients (left panel). Scale bars: upper panels=50 µm; lower panels=30 µm. Histograms display the mean ± SD of the percentage of PYGO2-positive cells (*n* = 8 fields per condition) in FFPE tissue sections. Statistical significance (***p < 0.001) was determined by a two-tailed non-parametric t-test (Mann-Whitney). **F** The scatter plot illustrates the relationship between *PYGO2* mRNA expression and *PYGO2* locus copy number in the CoMMpass dataset. Each point represents a single 1q21+ MM patient (*n* = 654). Spearman correlation between the two variables (*ρ* = 0.39, *p* < 2.2e-16). **G** Plot of the frequency of *PYGO2* top quartile (Q1) *vs* other quartiles (Q2–Q4) across CoMMpass genomic features (amp 1q, del 13q, del 17p, del 1p, gain 1q, gain 8q, hyperdiploidy, TP53 mutations, t(11;14), t(4;14)). Statistical analysis was performed using Fisher’s exact test, and q-values were adjusted for multiple testing with the Benjamini–Hochberg method. **H, I** Kaplan–Meier plot of progression-free survival (PFS) and overall survival (OS) according to *PYGO2* expression in CoMMpass dataset. Patients were stratified into top quartile (Q1, *PYGO2*_top=1, *n* = 151) vs other quartiles (Q2–Q4, *PYGO2*_top=0, *n* = 464). Statistical significance was assessed using the log-rank test (PFS *p* = 0.0045 and OS *p* = 0.0011). The lower panel shows the number of patients in each group at different time points (months).

To identify potential gene targets on 1q chromosome, we used our database registered to the Gene Expression Omnibus (GEO) of the National Center for Biotechnology Information with the accession number GSE227907 [27]. The database provides the transcription profiles of the 29 primary CD138^+^ samples (11 SMM and 18 NDMM) associated with the pattern of the 1q21 FISH results, indeed, the copy number of the 1q21 region. Among the identified genes in this database (Table 1), we focused on *PYGO2*, which was upregulated in 1q21+ patients compared to patient controls (CTL) (*p* = 0.0008) (Fig. 1A). We also observed a positive correlation between *PYGO2* gene expression and copy number of the 1q21 region (*p* = <0.0001, r = 0.6738) (Fig. 1B).

**Table 1:** The most correlated genes with the 1q21 copy number.

	*P*-VALUE	CHR. POSITION
***APH1A***	1,00E-06	q21.2
***ATP8B2***	1,00E-06	q21.3
***ILF2***	1,00E-06	q21.3
***PRKAB2***	1,00E-06	q21.3
***TARS2***	1,00E-06	q21.1
***UBE2Q1***	1,00E-06	q21.2
***INTS3***	2,00E-06	q21.3
***OTUD7B***	2,00E-06	q21.3
***PSMD4***	2,00E-06	q21.3
***FLAD1***	3,00E-06	q21.3
***CHTOP***	4,00E-06	q21.3
***DENND4B***	4,00E-06	q21.3
***SCNM1***	4,00E-06	q21.3
***GPR89A***	5,00E-06	q21.1
***PSMB4***	6,00E-06	q21.3
***PIP5K1A***	7,00E-06	q21.3
***IL6R***	9,00E-06	q21.3
***RNF115***	1,00E-05	q21.1
***ARNT***	1,40E-05	q21.3
***PYGO2***	4,80E-05	q21.3

To validate this data, we performed *PYGO2* gene expression analysis on purified primary MM PCs in a validation patients’ cohort (*n* = 43), confirming the significant overexpression in 1q21+ patients compared to CTL patients (*p* = 0.0011) (Fig. 1C). Next, we stratified patients according to the copy number of the 1q21 region and observed a trend of *PYGO2* overexpression from 1q21+ gain to amp samples (Fig. 1D). Furthermore, we performed immunohistochemistry on bone biopsies of MM patients (*n* = 4). PYGO2 expression was assessed by semi-quantitative analysis based on the percentage of positive cells for PYGO2 protein stained in the nucleus. We observed a significantly higher nuclear protein share of PYGO2 in 1q21+ patients than in CTL patients (*p* = 0.0003) (Fig. 1E, a representative patient and quantification across patients).

Finally, we analyzed public and CoMMpass databases. The analysis conducted in the CoMMpass dataset confirmed the significant correlation between the gene expression of *PYGO2* and the copy number of the *PYGO2* locus among MM patients (*p* < 2.2e-16, *r* = 0.39) (Fig. 1F). Based on the data from the CoMMpass database, we also divided the patients into quartiles according to the expression of *PYGO2*, resulting in four groups: Q1 (high expression), Q2 (medium-high expression), Q3 (medium-low expression), and Q4 (low expression). Then, we analyzed the frequency of various features, including genetic alterations and FISH results, in Q1 compared to Q2-Q4.

We did not observe significant differences with del17p, del1p, gain8q, mut TP53, and we noted a significant increase in hyperdiploidy or t(11;14) in patients with low *PYGO2* expression. In contrast, significantly higher *PYGO2* expression was found in MM patients carrying the 13q loss and t(4;14) (Fig. 1G). The data confirmed the co-occurrence between 1q CNA and 13q loss and suggested unfavorable clinical behavior for these patients, consistent with published data in the literature [31].

Furthermore, to investigate whether the expression level of *PYGO2* correlates with the survival of MM patients, Kaplan-Meier analysis was used. We found that patients in the *PYGO2* Q1 group have significantly poorer PFS (*p* = 0.0045) and OS (*p* = 0.0011) than those with lower expression (Fig. 1H, I).

Together, these results indicate that *PYGO2* is upregulated in 1q21+ patients at both mRNA and protein levels, showing a significant trend with 1q21 CNA, and its higher expression is associated with reduced patients’ PFS and OS, compared with lower expression.

### Knockdown of *PYGO2* affects cell proliferation

**Fig. 2:**
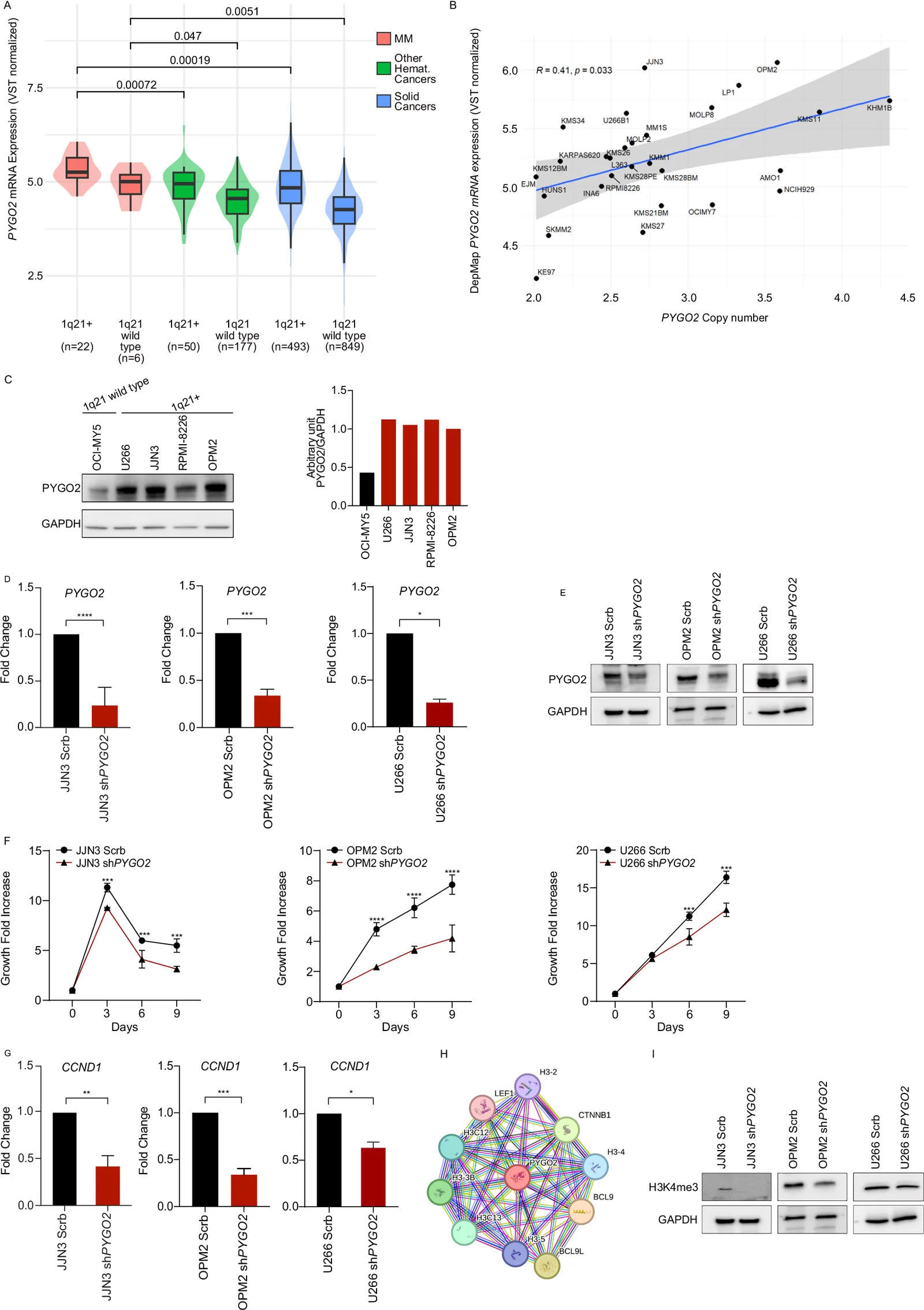
Knockdown of *PYGO2* affects cell proliferation. **A** The violin plot shows *PYGO2* mRNA expression across DepMap Public 24Q2 cell lines stratified by PYGO2 locus copy-number status: MM 1q21+ (*n* = 22) and 1q21 wild type (*n* = 6), other hematological cancers 1q21+ (*n* = 50) and 1q21 wild type (*n* = 177), solid cancers 1q21+ (*n* = 493) and 1q21 wild type (*n* = 849). Pairwise group comparisons were assessed with two-sided Wilcoxon rank-sum tests; *p*-values are annotated above the corresponding brackets. **B** The scatter plot shows the relationship between *PYGO2* mRNA expression and *PYGO2* locus copy number in HMCLs based on data derived from the DepMap database (*n* = 28). Each point represents an individual cell line (*PYGO2* mRNA expression, y-axis; 1q21 copy number, log2 transformed, x-axis). The association was evaluated using Spearman correlation (*ρ* = 0.41, *p* = 0.033). The gray region represents the 95% confidence interval around the linear regression line (blue). **C** PYGO2 protein expression in 1q21+ HMCLs (*n* = 4) and 1q21 wild-type cell line (*n* = 1) (left panel) by western blot. Densitometric quantification of PYGO2 protein in 1q21+ HMCLs and 1q21 wild-type cell line. The relative intensity of PYGO2 was normalized for the levels of GAPDH for each HMCL (right panel). **D** Fold change of *PYGO2* mRNA normalized to *GAPDH* mRNA control gene in JJN3 cells (left panel), OPM2 cells (middle panel), and U266 cells (right panel) after shRNA transduction by qRT-PCR. The experiments were performed at least three times. Statistical significance (*p < 0.05, ***p < 0.001) was determined by a two-tailed non-parametric t-test (Mann-Whitney). **E** PYGO2 protein expression in JJN3 cells, OPM2 cells, and U266 cell lines after shRNA transduction by western blot. **F** Effect of *PYGO2* loss in JJN3, OPM2, and U266 cells at three, six and nine days after shRNA selection on cell proliferation. The graphs represent three independent MTT assays performed in triplicate, calculated using JJN3, OPM2, and U266 Scrb as controls. Statistical significance (***p < 0.001, ****p < 0.0001) was determined by a two-tailed unpaired t-test. **G** Fold change of *CCND1* mRNA normalized to *GAPDH* mRNA control gene in JJN3 cells (left panel), OPM2 cells (middle panel), and U266 (right panel) after shRNA transduction by qRT-PCR. The experiments were performed at least three times. Statistical significance (*p < 0.05, **p < 0.01, left panel and ***p < 0.001, right panel) was determined by a two-tailed unpaired t-test. **H** Analysis of PYGO2 PPI network. Number of nodes: 11; number of edges: 53; PPI enrichment *p*-value: 6.28e-07. **I** H3K4me3 protein expression in JJN3, OPM2 and U266 cell lines days after shRNA transduction by western blot.

Firstly, we analyzed the DepMap dataset, and the analysis showed higher expression of *PYGO2* mRNA levels in MM compared to other haematological cancers and solid cancers. In particular, after stratifying by *PYGO2* locus copy number, MM lines with 1q21+ expressed more *PYGO2* than MM lines without 1q21 (Fig. 2A). Furthermore, MM cell lines still showed higher expression of *PYGO2* compared to other haematological tumors and solid tumors within the corresponding copy number strata (1q21+ and 1q21 wild type), indicating specific upregulation in MM (*p*-values annoted in Fig. 2A). These data revealed MM-specific upregulation of *PYGO2*, supporting the hypothesis of *PYGO2* involvement in MM.

Moreover, we observed an enhancement in *PYGO2* mRNA levels with increasing PYGO2 locus copy number (*p* = 0.017, *r* = 0.56) (Fig. 2B). Consistent with this analysis, we confirmed by WB the higher PYGO2 protein levels in several 1q21+ HMCLs (Table S2) compared to the OCI-MY5 line, which was used as a control as wild type for the 1q21 region. (Fig. 2C).

Then, we performed *PYGO2* knockdown using a short hairpin RNA lentiviral vector (sh*PYGO2*) in JJN3, OPM2 and U266, three HMCLs carrying 1q21+ CNA. We examined the mRNA levels of *PYGO2* by qRT-PCR. sh*PYGO2* mRNA expression was significantly decreased after *PYGO2* knockdown in JJN3 (*p* = 0.0001), OPM2 (*p* = 0.0001) and U266 (*p* = 0.02) (Fig. 2D). The protein expression was also down-regulated, as confirmed by WB analysis (Fig. 2E).

Furthermore, knockdown of *PYGO2* significantly reduces cell growth in JJN3 sh*PYGO2*, OPM2 sh*PYGO2* and U266 sh*PYGO2* compared to respective Scrb controls, as shown in the cell growth curves over 9 days (Fig. 2F).

Based on this reduced cell proliferation and knowing that PYGO2 regulates the transcription of some downstream targets, including *CCND1*, by qRT-PCR, we evaluated the mRNA levels of *CCND1* and observed a significant reduction in HMCLs upon knockdown of *PYGO2* (Fig. 2G).

To investigate the protein-protein interactions (PPI) of PYGO2, we obtained information from the STRING database. Among the uncovered interactions, as expected, we found factors involved in the β-catenin pathway, of which PYGO2 is a part, and some histone H3 variants (Fig. 2H). We focused on histone H3K4me3 based on a previous study showing that the human PYGO2 protein could stimulate transcriptional activation by directly binding this H3 variant [32]. By WB analysis, we specifically observed a decreased protein expression of H3K4me3 in HMCLs following *PYGO2* knockdown (Fig. 2I). The interesting feature is that H3K4me3 is on the promoter of *CCND1* [32].

Collectively, these data suggest that PYGO2 can regulate the H3K4me3 level and, consequently, the CCND1 expression levels, affecting MM cell proliferation.

### Transcriptional signature associated with *PYGO2* knockdown

**Fig. 3:**
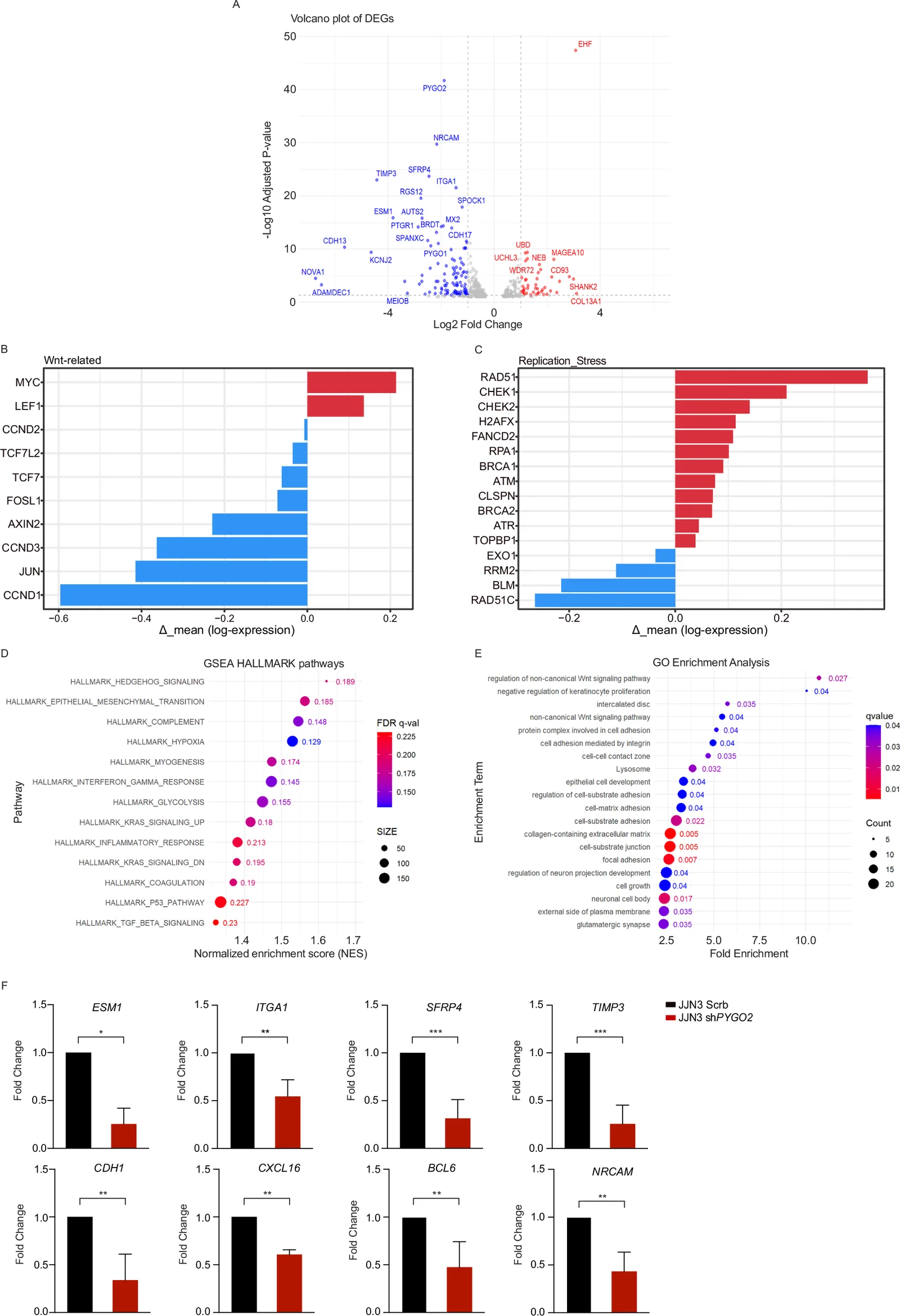
Transcriptional signature associated with *PYGO2* knockdown. **A** Volcano plot of RNA-Seq expression after *PYGO2* knockdown in JJN3. DEGs are depicted in blue if repressed (log2 fold change ≤ –1, Adj.*p* ≤ 0.05) or in red if upregulated (log2 fold change ≥ +1, Adj.*p* ≤ 0.05). **B** Differential expression of Wnt-related genes upon *PYGO2* knockdown in JJN3 MM cells. Bars show the mean expression difference between sh*PYGO2* and Scrb replicates (Δ=sh*PYGO2*−Scrb, log2-normalized RNA-seq units) for a curated Wnt target panel. Positive values indicate upregulation after *PYGO2* knockdown; negative values indicate downregulation. Replicates: *n* = 3 per condition. **C** Differential expression of replication-stress and DNA damage response (DDR) genes upon *PYGO2* knockdown in JJN3 MM cells. Bars show the mean expression difference between sh*PYGO2* and Scrb replicates (Δ=sh*PYGO2*−Scrb, log2-normalized RNA-seq units) for a curated panel. Positive values indicate upregulation after *PYGO2* knockdown; negative values indicate downregulation. Replicates: *n* = 3 per condition. **D** Dot plot showing the enrichment of hallmark gene sets in JJN3 Scrb compared to JJN3 sh*PYGO2* after *PYGO2* knockdown. Each dot represents a hallmark pathway, with its position on the x-axis indicating the normalized enrichment score (NES). The color gradient reflects the false discovery rate (FDR) q-value, while the dot size is proportional to the number of genes in each gene set. Gene Set Enrichment Analysis (GSEA) was performed using the Broad Institute GSEA desktop application (v4.4.0) with hallmark gene sets from the Molecular Signatures Database (MSigDB, v2024.1.Hs). Genes were ranked by signal-to-noise ratio, and enrichment was assessed using 10000 phenotype permutations. Statistical significance was determined using a permutation-based, non-parametric test employing a Kolmogorov–Smirnov-like statistic, as implemented in the GSEA software. Normalized enrichment scores (NES) and FDR q-values were used to evaluate pathway significance. **E** Dot plot showing the Gene Ontology (GO) biological process enrichment analysis in JJN3 Scrb compared to JJN3 sh*PYGO2* after *PYGO2* knockdown. Each dot represents a GO enrichment term, with its position on the x-axis indicating the Fold enrichment score. Enrichment analysis was performed with the gseGO function from the ClusterProfiler R package using default settings. The x-axis represents fold enrichment, the y-axis lists enriched GO terms, the color gradient indicates q-values, and the dot size corresponds to the number of genes in each category. **F** Fold change of *ESM1, ITGA1, SFRP4, TIMP3, CDH1, CXCL16, BCL6 and NRCAM* mRNA normalized to *GAPDH* mRNA control gene in JJN3 cells after shRNA transduction by qRT-PCR. The experiments were performed at least three times. Statistical significance (*p < 0.05, **p < 0.01, ***p < 0.001) was determined by a paired t-test.

Since the role of *PYGO2* in MM has not been defined, we performed RNA-seq analysis on JJN3 Scrb and JJN3 sh*PYGO2* to identify DEGs related to *PYGO2* knockdown and possible pathways linked to the 1q21+ cell unfavorable features. We identifed 452 differentially expressed genes (DEGs) (*p* < 0.05), among which 143 genes were upregulated and 309 downregulated in JJN3 sh*PYGO2*, as shown in Volcano plot (Fig. 3A).

To investigate pathway-level changes downstream of *PYGO2*, we examined a curated panel of canonical WNT-related genes and quantified the mean expression difference between sh*PYGO2* and Scrb replicates. *PYGO2* knockdown reduced the expression of several hallmark Wnt/TCF targets involved in proliferation, including *CCND1*, *JUN*, *CCND3*, and *AXIN2*, and also decreased *FOSL1*, with modest reductions in *TCF7*. In contrast, *MYC* and *LEF1* showed a slight increase (Fig. 3B). We next assessed replication-stress and DNA damage response (DDR) gene sets using the same metric. *PYGO2* knockdown in JJN3 cells produced a shift in the replication-stress/DDR transcriptome, with a general trend toward increased expression of checkpoint and genes associated with damage detection, such as *RAD51*, *CHEK1*, *CHEK2*, *H2AFX*, and *FANCD2*. In parallel, selected homologous recombination and genes involved in nucleotide synthesis showed reduced expression (Fig. 3C). Overall, these changes suggest a tendency toward elevated replication stress.

In addition, GSEA [33] based on MSigDB Hallmark gene sets [34] showed that hedgehog signaling, epithelial-mesenchymal transition, complement signaling, interferon-γ response signaling, inflammatory response signaling and TGF-β signaling were enriched in JJN3 Scrb (FDR *q* value < 0.25), i.e., in a cell line with higher *PYGO2* expression than the sh*PYGO2* cell line (Fig. 3D). These results of RNA-seq analysis could suggest that *PYGO2* also performs an immunomodulatory function, as previously demonstrated in prostate cancer [35].

Notably, Gene Ontology (GO) biological process (BP) analysis indicated that some BPs, such as cell growth, cell-substrate adhesion, cell-matrix adhesion, and cell adhesion mediated by integrin pathways, were significantly enriched in cells with *PYGO2* knock-down (Fig. 3E). Therefore, these RNA-seq data are in line with the data above produced in vitro, as the decreased cell proliferation following down-regulation of *PYGO2*. Finally, several genes are validated by qRT-PCR (Fig. 5F). Some of these genes are shared by BPs, i.e., *BCL-6* is shared among cell growth, cell-substrate adhesion and cell-matrix adhesion. *ITGA1* is related to cell adhesion mediated by integrin, *CXCL16* and *NRCAM* are specific for cell growth.

Together, the data obtained from RNA-seq analysis confirmed an involvement of *PYGO2* in cell proliferation and its possible role in immune modulation in MM.

### The impact of *PYGO2* knockdown on the cell cycle

**Fig. 4:**
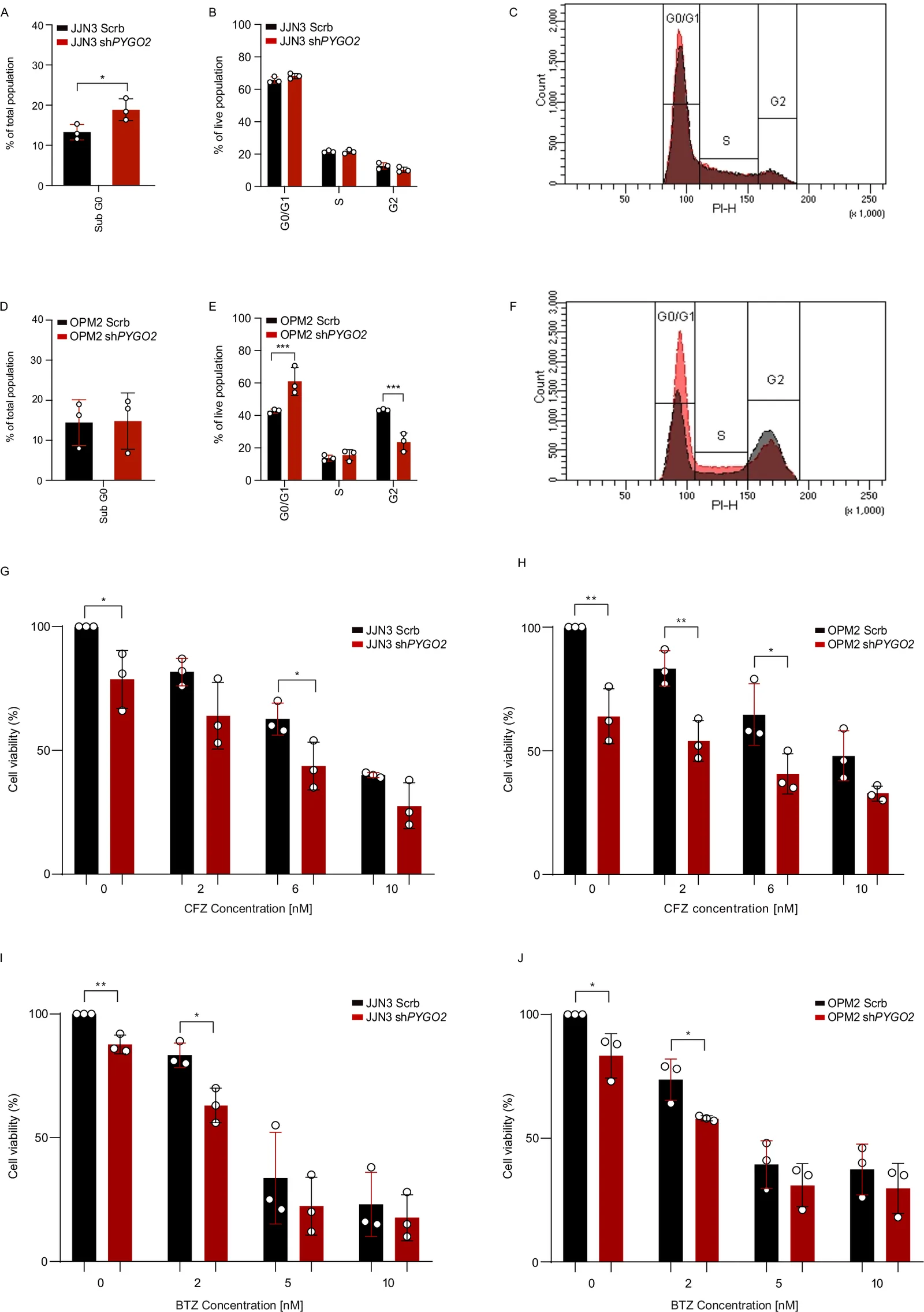
*PYGO2* knockdown affects the cell cycle and makes MM cells more sensitive to CFZ and BTZ treatment. **A, D** Effect of *PYGO2* knockdown on the Sub G0 fraction of the cell cycle using shRNA in JJN3 and OPM2 in three independent experiments by flow cytometry assay. Fxcycle TMPI/RNase staining of MM cells was conducted at 72 h. A minimum of 100,000 events was collected for each condition. Statistical significance (*p < 0.05) was determined by a two-tailed unpaired t-test. **B, E** Effect of *PYGO2* knockdown using shRNA on cell cycle in JJN3 and OPM2 (live population) in three independent experiments by flow cytometry assay. Fxcycle TMPI/RNase staining of MM cells was conducted at 72 h. A minimum of 100,000 events was collected for each condition. Statistical significance (***p < 0.001) among groups was determined by 2-way ANOVA using Sidak’s correction for multiple comparison testing. **C, F** Overlay of a representative analysis of the cell cycle of the JJN3 and OPM2 sh*PYGO2* line compared to JJN3 and OPM2 Scrb. **G, H** JJN3/OPM2 sh*PYGO2* and Scrb were treated with different concentrations of CFZ (2 nM, 6 nM, 10 nM) for 48 h. The graph represents the cell viability (± SD) of three independent experiments performed in triplicate, calculated using JJN3/OPM2 Scrb as a control. Statistical significance (*p < 0.05, **p < 0.01) was determined by an unpaired t-test. **I, J** JJN3/OPM2 sh*PYGO2* and Scrb were treated with different concentrations of BTZ (2 nM, 5 nM, 10 nM) for 48 h. The graph represents the cell viability (± SD) of three independent experiments performed in triplicate, calculated using JJN3/OPM2 Scrb as a control. Statistical significance (*p < 0.05, **p < 0.01) was determined by an unpaired t-test.

Given the key role of CCND1 in the cell cycle, we performed a cell cycle analysis using propidium iodide staining and flow cytometry in JJN3/OPM2 Scrb and sh*PYGO2* at 72 h. In JJN3 cell line, the *PYGO2* knockdown significantly increased the Sub G0 cell fraction compared to the control, indicating an enhanced cell death in JJN3 sh*PYGO2* (Fig. 4A). The distribution of cells across the G0/G1, S, and G2 phases did not exhibit substantial differences between the two conditions, suggesting that *PYGO2* knockdown did not significantly impact cell cycle progression concerning the live population (Fig. 4B, C, quantification across experiments and overlay of a representative analysis, respectively). On the other hand, the cell cycle analysis of the OPM2 cell line revealed a different trend. Specifically, OPM2 sh*PYGO2* did not exhibit differences in the Sub G0 population compared to OPM2 Scrb (Fig. 4D). Interesting data emerged on the live population. In fact, OPM2 sh*PYGO2* showed a significant increase in the G0/G1 phase population compared to the control, accompanied by a significant reduction in the G2 phase population, while the percentage of cells in the S phase remained unchanged between the two conditions (Fig. 4E, F, quantification across experiments and overlay of a representative analysis, respectively). Thus, *PYGO2* knockdown results in an increased Sub-G0 fraction in JJN3 and in G0/G1 phase cell cycle arrest in the OPM2 cell line. Taken together, these data suggest that *PYGO2* plays a crucial role in the cell cycle.

Because recent data indicate that the presence of 1q21+ is associated with a reduced response to PIs, including CFZ and BTZ [8, 10], we investigate the possible role of *PYGO2* in the in vitro response of MM cells to CFZ and BTZ. To elucidate whether *PYGO2* could influence treatment with both PIs, we treated sh*PYGO2* cell lines with different concentrations of CFZ and BTZ for 48 h. Indeed, cells with sh*PYGO2* showed significantly higher mortality treated with CFZ and BTZ than the Scrb cell lines (Fig. 4G–J), suggesting a possible role in increasing PIs sensitivity of MM cells.

### Overexpression of *PYGO2* increases MM cell proliferation

**Fig. 5:**
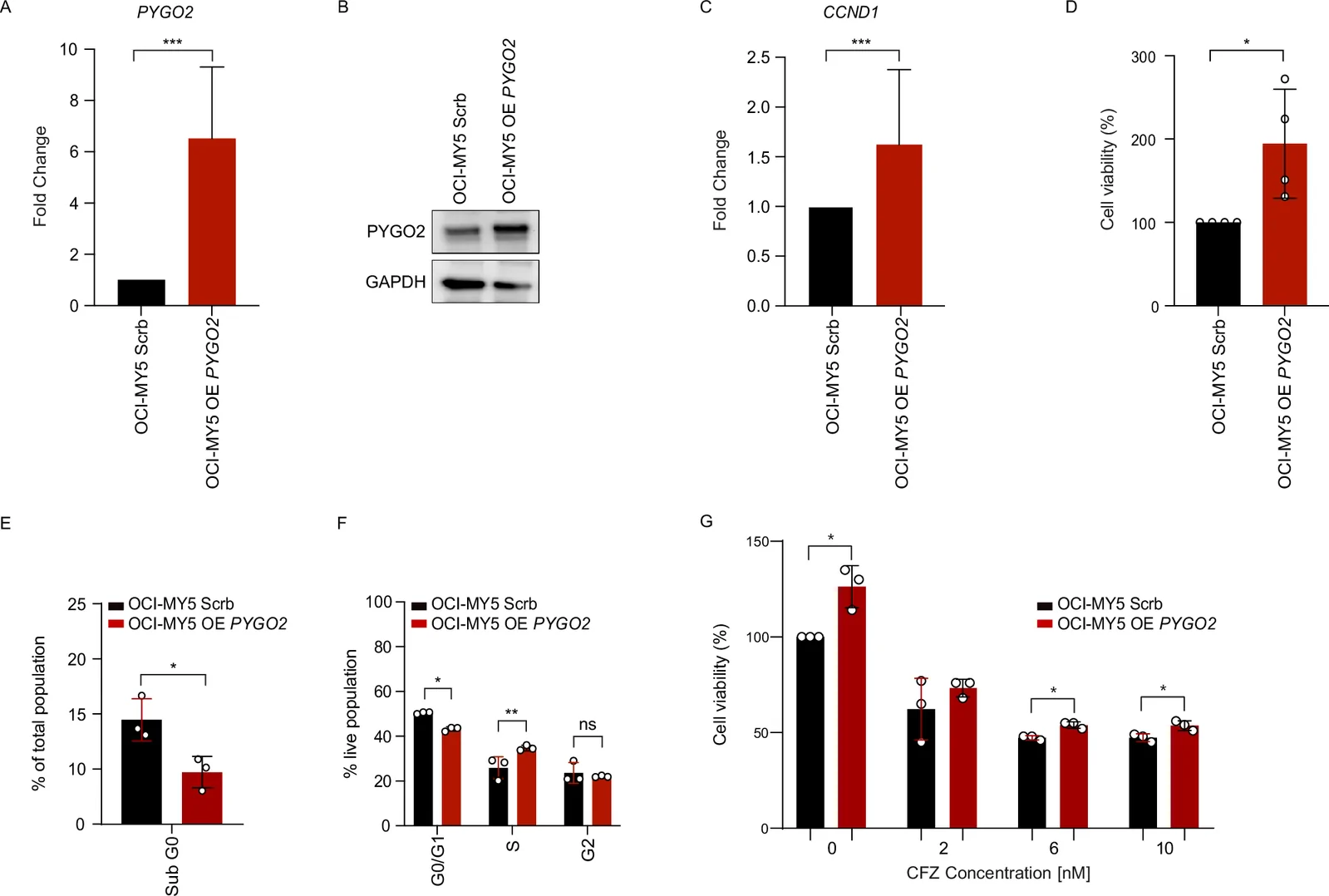
Overexpression of *PYGO2* increases MM cell proliferation. **A** Fold change of *PYGO2* mRNA normalized to *GAPDH* mRNA control gene in OCI-MY5 cells after OE *PYGO2* transduction by qRT-PCR. The experiments were performed at least three times. Statistical significance (***p < 0.001) was determined by a two-tailed non-parametric t-test (Mann-Whitney). **B** PYGO2 expression in OCI-MY5 after *PYGO2* OE transduction by western blot. **C** Fold change of *CCND1* mRNA normalized to *GAPDH* mRNA control gene in OCI-MY5 cells after *PYGO2* OE transduction by qRT-PCR. The experiments were performed at least three times. Statistical significance (***p < 0.001) was determined by a two-tailed non-parametric t-test (Mann-Whitney). **D** The graphs represent the percentage of cell viability of four independent MTT tests performed in triplicate, calculated using OCI-MY5 Scrb as a control. Statistical significance (*p < 0.05) was determined by an unpaired t-test. **E, F** Effect of OE *PYGO2* on the Sub G0 fraction and on cell cycle (live population) in OCI-MY5 after OE *PYGO2* transduction in three independent experiments by flow cytometry assay. Fxcycle TMPI/RNase staining of MM cells was conducted at 72 h. A minimum of 100,000 events was collected for each condition. Statistical significance (*p < 0.05, **p < 0.01) was determined by an unpaired t-test. **G** OE *PYGO2* and Scrb were treated with different concentrations of CFZ (2 nM, 6 nM, 10 nM) for 48 h. The graph represents the cell viability (± SD) of three independent experiments performed in triplicate, calculated using OCI-MY5 Scrb as a control. Statistical significance (*p < 0.05) was determined by an unpaired t-test.

To confirm that *PYGO2* is significantly involved in the MM cell growth regulation, we overexpressed *PYGO2* in OCI-MY5, the wild-type cell lines for the 1q21+ region. We verified the significant OE *PYGO2* on OCI-MY5 through qRT-PCR and WB (Fig. 5A, B, respectively). We further confirmed the subsequent significant upregulation of *CCND1* following the OE *PYGO2* (Fig. 5C). Then, we evaluated the cell proliferation, and we showed a significant increase in cell proliferation of OCI-MY5 OE-*PYGO2* compared to the Scrb control (Fig. 5D).

Subsequently, as done for sh*PYGO2*, we performed the cell cycle analysis on OCI-MY5 OE *PYGO2*. Our experiments revealed that OE *PYGO2* widely influences the progression of the cell cycle in MM cells, leading to a significant reduction of cells in the Sub G0 fraction (Fig. 5E). Moreover, among live population, we found a significant decrease in the fraction of cells distributed in the G0/G1 phase, a consistent significant increase of cells in the S phase, with no substantial change in the G2 (Fig. 5F).

Subsequently, we performed an MTT assay to assess the CFZ treatment response in cells overexpressing *PYGO2*. We observed a reduced response to CFZ treatment at different concentrations (Fig. 5G).

Overall, these results suggest that *PYGO2* overexpression supports the vitality and growth of MM cells.

## Discussion

In this study, we identified *PYGO2* as a gene located on the 1q21 chromosomal region whose expression is significantly increased in CD138^+^ PCs of MM patients with 1q21+, in a copy number-driven fashion. Our results indicate that *PYGO2* expression correlates with gain/amp of 1q21+ CNA, is associated with poor prognosis, and plays a functional role in MM cell proliferation and cell cycle regulation.

1q21+ CNA is one of the most frequent cytogenetic abnormalities in MM, and it is associated with shorter progression-free survival [10]. For this reason, identifying new mechanisms underlying these unfavorable features and a possible new drug target in the 1q21 region is very important for patients with 1q21+ MM. Our results support the hypothesis that *PYGO2* could be a biomarker for aggressive MM, even though several genes have previously been identified as candidate genes related to 1q21+ [14, 27].

We first demonstrated that *PYGO2* is overexpressed at both mRNA and protein levels in 1q21+ MM patients compared to MM patients without 1q21+. This overexpression was also observed in several independent datasets, including GSE227907 and the CoMMpass dataset, where *PYGO2* expression showed a strong positive correlation with the number of 1q21 copies. In addition to these datasets, we also investigated the DepMap dataset, and we demonstrated the overexpression of *PYGO2* in MM compared to other hematological malignancies, to highlight the impact of 1q21+ in MM. Furthermore, high *PYGO2* expression was associated with adverse cytogenetic features, such as del(13q) or t(4;14), and significantly shorter progression-free and overall survival, underscoring its clinical relevance.

PYGO2 is a nuclear chromatin reader and Wnt/β-catenin transcriptional co-activator composed of an NHD domain and PHD finger. Within the β-catenin/TCF complex, PYGO2 links the transcriptional machinery to H3K4-methylated active chromatin; its PHD finger recognizes H3K4me2/3 and forms a composite interface with the HD1 domain of the adaptor BCL9/BCL9L, while the HD2 domain of BCL9 engages β-catenin. Through this tripartite β-catenin-BCL9-PYGO2 assembly, the β-catenin/TCF complex is anchored to H3K4me3-enriched Wnt response elements, stabilizing promoter occupancy and enhancing transcription of canonical targets [21, 22, 32, 36, 37].

Functionally, *PYGO2* knockdown in 1q21+ MM cell lines led to a marked reduction in proliferation, at least partially, through downregulation of *CCND1*, a key regulator of G1/S transition. Mechanistically, we observed that *PYGO2* knockdown reduced the levels of H3K4me3, present on the promoter of CCND1. We acknowledge that in MM, including the cell lines analyzed in our study, *CCND2* represents the predominant D-type cyclin. However, we focused on *CCND1* because it is a well-established downstream target of the Wnt pathway, and PYGO2 cooperates with β-catenin to modulate *CCND1* transcription. For this reason, *CCND1* provides a mechanistically relevant readout of *PYGO2*-dependent Wnt activity. These findings are consistent with previous reports in human brain glioma [32], showing that PYGO2 can bind H3K4me3, thereby modifying target gene expression.

RNA-seq analysis of cells with *PYGO2* knockdown revealed the modulation of multiple transcriptional programs relevant to MM biology. Beyond the attenuation of Wnt proliferative outputs, as previously reported [36], *PYGO2* knockdown was associated with transcriptional signatures indicative of increased replication stress and activation of checkpoint/DDR pathways, suggesting that *PYGO2* contributes to both proliferative competence and genome-stability maintenance. In parallel, *PYGO2* knockdown downregulates multiple oncogenic pathways, including interferon-γ response and TGF-β signaling, alongside enrichment in gene sets involved in cell adhesion and integrin signaling. These findings suggest that PYGO2 may not only regulate cell proliferation but also modulate tumor-microenvironment interactions and immune signaling. Such a role has been previously proposed in prostate cancer [35]. Although this aspect remains unexplored in MM, it opens interesting avenues for future research into the immunomodulatory potential of *PYGO2* targeting.

Interestingly, cell cycle analysis revealed distinct effects of PYGO2 depletion in different MM cell lines. In JJN3 cells, *PYGO2* knockdown induced a significant increase in Sub G0 population, whereas in OPM2 cells, it led to G1 and G2 arrest. This suggests that the functional consequences of *PYGO2* knockdown may be MM line-dependent. In the JJN3 cell line, the transcriptional upregulation of checkpoint and DNA damage response genes reveals activation of a DNA damage surveillance program upon *PYGO2* knockdown. This is consistent with the observed increase in the Sub G0 fraction, indicating that knockdown of *PYGO2* in the JJN3 cell line induces genotoxic stress, possibly leading to the elimination of a subset of cells while leaving cell-cycle progression largely unaffected in the surviving population. In this regard, the fact that the JJN3 cell line harbours an *MAF* translocation, which is known to establish dependencies on D-type cyclins other than *CCND1*, may contribute to the cell-cycle response observed in this line upon *PYGO2* knockdown.

Conversely, overexpression of *PYGO2* in a 1q21 wild-type MM cell line (OCI-MY5) resulted in increased *CCND1* expression, accelerated cell cycle progression, and enhanced proliferation, further supporting the role of PYGO2 as a driver of MM cell growth. Furthermore, treatment with PIs showed enhanced cell sensitivity following *PYGO2* knockdown and reduced response in OCI-MY5, upon *PYGO2* overexpression, as if *PYGO2* protected MM cells from the PI effect.

A recently published functional‑genomics study further highlighted the marked heterogeneity of dependency patterns in MM [15]. In particular, while some genes, such as *MCL1*, display strong and uniform dependency across models [15], others show more context‑specific roles, as is the case in our study. Within this framework, our data indicate that *PYGO2* does not elicit a uniformly strong dependency across MM cell lines, as illustrated by the reduction of a proliferative rate in JJN3 and U266 cells upon *PYGO2* knockdown. In contrast, OPM2 cells show a clear reduction in proliferation following *PYGO2* knockdown, highlighting a model-specific requirement for *PYGO2*. These differences indicate that the function of *PYGO2* varies depending on the transcriptional context and oncogenic drivers characteristic of each MM subtype. Importantly, even in settings where *PYGO2* does not impose a strict proliferative dependency, our transcriptomic analyses reveal consistent modulation of Wnt-related programs and replication-stress responses, supporting a biologically relevant and context-dependent role for *PYGO2* in MM.

Our data collectively support the hypothesis that PYGO2 acts as a previously unrecognized effector of 1q21-driven disease progression in MM. The functional dependence of 1q21+ MM cells on PYGO2 raises the possibility that it may represent a druggable target for 1q21+ MM patients. Specifically, efforts to disrupt its interaction with the Wnt transcriptional machinery or its chromatin-binding domains could provide a basis for a therapeutic strategy. For instance, the development of small-molecule inhibitors specifically targeting *PYGO2* could be an attractive approach.

In conclusion, we demonstrate for the first time the *PYGO2* overexpression in MM patients with 1q21+, its association with worse survival outcomes, and its functional role in promoting tumor vitality and proliferation. These results suggest that PYGO2 could be a target for a personalized approach in the subgroup of MM patients with 1q21+.

## Supplementary information

Methods Supplementary

Tables Supplementary

## Data Availability

All data generated or analyzed during this study are included in this published article and its supplementary information files. For original data, please contact nicola.giuliani@unipr.it. The data generated in this study are available upon request from the corresponding authors. These data were generated as part of the Multiple Myeloma Research Foundation Personalized Medicine Initiatives (https://research.themmrf.org and www.themmrf.org), and the CoMMpass data are available upon registration in the MMRF Researcher Gateway at https://research.themmrf.org.
