# Supplementary material for: PYGO2 is overexpressed in multiple myeloma patients with 1q21 amplification, driving plasma cell proliferation and cell cycle progression
Source: Cancer Gene Ther. 2026 Jul 11;33(8):919–30. doi: 10.1038/s41417-026-01060-5 (PMC13498461; doi:10.1038/s41417-026-01060-5)
Supplement: Supplementary file 1 [file 41417_2026_1060_MOESM1_ESM.pdf]

Methods Supplementary

Fluorescence in Situ Hybridization (FISH)

Cell suspensions (5×10^5–1.5×10^6 cells) were incubated in a hypotonic KCl solution (0.075 M) for 8–10 minutes and then fixed in Carnoy's fixative (3:1 methanol:glacial acetic acid). Fixed cells were spread onto glass slides and dehydrated through three ethanol concentrations (70%, 85%, 100%) for 2 minutes each. The slides were then treated with 10 µl of the XL *CDKN2C/CKS1B* probe (MetaSystems, Altussheim, Germany). DNA denaturation and probe codenaturation were performed at 75 ± 1°C for 5 minutes, followed by hybridization at 37°C for 12–16 hours. Post-hybridization washes were conducted with 0.4×SSC/0.3% NP-40 at 73 ± 1°C and 2×SSC/0.1% NP-40 at room temperature. Slides were counterstained with DAPI (10 µl) and examined under a fluorescence microscope. The XL *CDKN2C/CKS1B* probe, labeled with Spectrum Orange (1q21) and Spectrum Green (1p32), detects deletions or amplifications in the *CKS1B* and *CDKN2C* loci.

Gene Expression Analysis

Biotin-labeled complementary RNA was prepared and hybridized to GeneChip ClariomD Arrays (Affymetrix, Thermo Fisher Scientific) according to the manufacturer's instructions. Arrays were scanned using the GeneChip Scanner 3000 7G (Affymetrix Inc.). Data analysis was performed in R (version 4.0.2) within RStudio (v1.3.959) using the affyQCReport package for quality assessment. Global expression data for 19,012 protein-coding genes and 13,972 long non-coding RNAs were extracted, normalized, and annotated based on Gencode v26 (University of Michigan) and updated with Ensembl v102 via the biomaRt package. Regional amplification was assessed using loess smoothing of the W statistic and p-value significance between 1q-amplified and wild-type samples. Differential expression was analyzed with 1 million permutations and a q-value of 0 to control the false discovery rate. The correlation of amplified nuclei (out of 100 screened) was evaluated using linear regression with the global test function in R.

RNA Isolation, Reverse Transcriptase PCR Amplification and Real-Time Quantitative PCR

Total RNA was extracted from patient samples and HMCLs using the RNeasy Kit (Qiagen), quantified with a NanoDrop ND-100 (Celbio) and suspended in 350 µl RLT/β-mercaptoethanol buffer. The RNA was precipitated and re-suspended in RNAse and DNAse free water. 1 µg was reverse-transcribed using the MULV

Reverse Transcriptase Kit (Life Technologies). qRT-PCR was performed on a LightCycler 480 (Roche) using the Universal Probes Master Mix and TaqMan assays for:

PYGO2 (Hs00540767_m1)

CCND1 (Hs00765553_m1)

ESM1 (Hs001199831_m1)

ITGA1 (Hs00235006_m1)

SFRP4 (Hs00180066_m1)

TIMP3 (Hs00165949_m1)

NRCAM (Hs01031598_m1)

CDH1 (Hs01023894_m1)

CXCL16 (Hs00222859_m1)

BCL6 (Hs00153368_m1)

SPOCK1 (Hs00270274_m1)

GAPDH (Hs99999905_m1)

B2M (Hs00187842_m1)

Reactions were run in duplicate, and relative expression was calculated using the comparative Ct method with *GAPDH* or *B2M* as endogenous controls.

Human Myeloma Cell Lines (HMCLs)

HMCLs (OCI-MY5, JJN3, U266, OPM2, and RPMI-8226) were obtained from DSMZ (Braunschweig, Germany) and cultured in RPMI 1640 medium supplemented with 20% fetal bovine serum (FBS), penicillin (100 U/ml), L-glutamine (2 nM), and fungizone (2.5 µg/ml) (all from Gibco Invitrogen, Milan, Italy).

Immunohistochemistry and immunoblotting

Hematoxylin and eosin (H&E) and immunohistochemical (IHC) staining were performed on formalin-fixed paraffin-embedded (FFPE) bone biopsies from 4 MM patients. The primary antibody employed for the study is PYGO2 Rabbit (DBA, 1:500 dilution). All reactions were revealed using an UltraView Universal DAB Detection Kit (Ventana, Export, PA, USA) or IHC Detection Kit-Micropolymer (Abcam, Cambridge, UK; ab236466) according to the manufacturer's recommendations. Light hematoxylin was used to counterstain nuclei. Negative controls consisted of sections exposed to the same procedure but omitting primary antibodies.

Regarding immunoblotting, the whole cell lysate was obtained using a commercial kit (Active Motif, Carlsbad, CA, USA) following the manufacturer's protocol. For immunoblotting, the following antibodies were used: PYGO2 (AF3616) Goat polyclonal Ab (1:500 dilution, R&D Systems, Inc.), GAPDH (14C10) Rabbit mAb (1:1000 dilution, Cell Signaling Technology), H3K4me3 (ab8580) Rabbit (1:1000 dilution, Abcam). Protein bands were quantified using ImageJ software (U.S. National Institutes of Health, Bethesda, MA, USA).

Cells Viability Assay (MTT)

The MTT assay (Sigma-Aldrich, 3050 Spruce Saint Louis, MO 63103) was used to determine the cellular viability of JJN3 and OPM2 infected with sh*PYGO2* scramble and sh*PYGO2*. In brief, 10.000 cells/well were seeded in 90 µl of culture medium. Regarding the treatment with CFZ, we used 90 µl of culture medium containing the appropriate drug concentration (6nM, 8nM, 10nM). Incubate cell cultures for 72 hours at +37°C and 5% CO2. After incubating, add 10 µl of MTT labelling reagent to each well. Incubate the microplate for 2 hours in a humidified atmosphere (37°C, 5% CO2). The viability of the cells was evaluated by spectrophotometry (ThermoScientific, Multiskan FC, type: 357).

Drugs: The Carfilzomib (CFZ) was purchased by Bio-Techne SRL; Bortezomib (BTZ) was purchased by Selleckchem.

Cell Cycle Assay

Cell Cycle was assessed using BD FACSAria™ flow cytometer (BD Bioscience, Italy) and FxCycle™ Propidium Iodide/RNase Staining Solution. Briefly, samples for cell cycle analysis were fixed in 70% EtOH at 4 °C for at least 2 hours and incubated with FxCycleTM Propidium Iodide/RNase Staining Solution (Life Technologies) for 30 min protected from the light, according to the manufacturer's instructions. Samples were

analyzed using a 488-nm, 532-nm or similar excitation, and collecting the emission using a 585/42 bandpass filter or equivalent.
