# Supplementary material for: PYGO2 is overexpressed in multiple myeloma patients with 1q21 amplification, driving plasma cell proliferation and cell cycle progression
Source: Cancer Gene Ther. 2026 Jul 11;33(8):919–30. doi: 10.1038/s41417-026-01060-5 (PMC13498461; doi:10.1038/s41417-026-01060-5)
Supplement: Supplementary file 2 [file 41417_2026_1060_MOESM2_ESM.pdf]

Table 1. Cytogenetic features of our validation cohort of patients. Schematization of some patient characteristics: samples, diagnosis, presence of 1q21+, FISH pattern, gender and age.

SAMPLES	DIAGNOSIS	1q21+	PATTERN FISH	GENDER	AGE
MM1	NDMM	No	2G2O	F	77
MM2	NDMM	No	2G2O	F	80
MM3	NDMM	No	2G2O	F	82
MM4	NDMM	No	2G2O	F	85
MM5	NDMM	No	2G2O	F	72
MM6	NDMM	No	2G2O	M	49
MM7	NDMM	No	2G2O	M	41
MM8	NDMM	No	2G2O	M	61
MM9	NDMM	No	2G2O	M	79
MM10	NDMM	No	2G2O	M	79
MM11	NDMM	Amp	58n:3G4O, 42n:2G3O	M	89
MM12	NDMM	Gain	32n:2G2O, 168n:2G	M	72
MM13	NDMM	Gain	60n:2G2O, 40n:2G3O	F	86
MM14	NDMM	Gain	93n:1G2O, 7n:1G3O	M	81
MM15	NDMM	Gain	162n:1G3O, 19n:1G2O, 8n:1G4O, 7n:2G2O	F	60
MM16	NDMM	Amp	11n:2G2O, 2n:1G1O, 5n:0G2O, 83n:0G6O	M	83
MM17	NDMM	Amp	176n:2G4O, 20n:2G3O, 4n:2G2O	F	45
MM18	NDMM	Amp	1G3O, 2G5/6/7O	F	57
MM19	NDMM	Gain	66n:2G2O, 44n:2G3O	F	71
MM20	NDMM	Gain	75n:2G3O, 25n:2G2O	F	79
MM21	NDMM	Gain	82n:2G2O, 18n:2G3O	F	87
MM22	NDMM	Gain	75n:2G3O, 25n:2G2O	M	74
MM23	NDMM	Amp	122n:2G4O, 54n:2G3O, 6n:2G5O, 18n:2G2O	M	59
MM24	NDMM	Amp	170n:2G4O, 14n:2G3O, 6n:4G8O, 10n:2G5/6O	F	63
MM25	RMM	No	2G2O	M	66
MM26	RMM	No	2G2O	F	72
MM27	RMM	No	2G2O	M	74
MM28	RMM	No	2G2O	F	84
MM29	RMM	Gain	62n.2G3O 15n:4G6O 10n:2G2O 3n:4G4O	F	75
MM30	RMM	Gain	62n0G3O, 34n0G2O, 6n0G4O	F	73
MM31	RMM	Gain	85n:2G3O 15n:2G2O	F	76
MM32	RMM	Amp	2G4O	F	56
MM33	RMM	Gain	72n:2G3O, 27n:2G2O	F	75
MM34	RMM	Gain	64n:2G3O, 16n:2-4G6O 8n:2G4O, 12n:2G2O	F	68
MM35	RMM	Gain	154n:1G3O, 23n:1G4O, 23n:1G2O	M	74

MM36	RMM	Amp	70n:2G6O, 24n:2G5O, 6n:2G2O	M	78
MM37	RMM	Amp	118n:1G4O	M	75
MM38	RMM	Gain	5n:2G2O, 95n:2G3O	M	60
MM39	RMM	Gain	78n:1G3O, 10n:2G3O, 12n:2G2O	F	82
MM40	RMM	Amp	28n:2G2O, 37n:2G4O, 24n:2G3O, 8n:5O2G	M	84
MM41	RMM	Amp	73n:2G4O, 89n:2G5O, 6n:2G6O, 14n:2G8O, 18n:2G2O	M	65
MM42	RMM	Amp	42n:2G4O, 6n:2G5O, 41n:2G3O, 12n:2G2O	M	79
MM43	RMM	Amp	84n:2G4O, 6n:4G8-10, 6n:2G3O, 4n:2G2O	F	83

Table 2. FISH pattern characteristic of each HMCLs performed in this study.

MM cell lines	1q21 copy number (SG 1p32; SO 1q21)
OCI-MY5	2G20(100%)
JJN3	3G4O(80%); 3G5O(14%); 6G8O(6%)
U266	3G4O(90%)
OPM2	3G6O(15%); 3G7O(31%); 3G8O(53%)
RPMI-8226	2G5O(78%); 2G4O(22%)
